# Regulatory effects of zinc on cadmium-induced cytotoxicity in chronic inflammation

**DOI:** 10.1371/journal.pone.0180879

**Published:** 2017-07-25

**Authors:** Paola Bonaventura, Aline Lamboux, Francis Albarède, Pierre Miossec

**Affiliations:** 1 Department of Immunology and Rheumatology, Immunogenomics and inflammation research Unit EA 4130, University of Lyon, Edouard Herriot Hospital, Lyon, France; 2 Geology Laboratory–Department of Earth Sciences, Ecole Normale Supérieure de Lyon and CNRS Lyon, Lyon, France; Centre National de la Recherche Scientifique, FRANCE

## Abstract

**Objectives:**

Zinc (Zn) has major effects on immune system activation while Cadmium (Cd) has anti-inflammatory and anti-proliferative effects in several chronic inflammatory contexts. The aim of this work was to investigate by which mechanisms Zn could compete with Cd and eventually counteract its deleterious effects. Rheumatoid arthritis (RA) synoviocytes exposed to cytokines were used as a model of chronic inflammation; osteoarthritis (OA) synoviocytes were used as control.

**Methods:**

Cell/medium fractionation constants were analyzed for different metals by inductively-coupled-plasma mass-spectrometry by comparison to the ^70^Zn spike. Interleukin-17 (IL-17) and tumor necrosis factor-alpha (TNF-α) were used to mimic inflammation. Gene expression of ZIP-8 importer, metallothioneins-1 (MT-1s) and the ratio between metalloprotease-3 and the tissue inhibitor of metalloproteinases (MMP-3)/TIMP-1) were evaluated after pre-exposure to cytokines and Cd, with or without the addition of exogenous Zn (0.9 ppm). Cell viability was measured by neutral red assay and IL-6 production by ELISA.

**Results:**

Synoviocytes selectively absorbed and retained Cd in comparison to Zn. Metal import increased with IL-17/TNF-α exposure, through the enhanced ZIP-8 expression. Zn did not modify ZIP-8 expression, while Cd reduced it (p<0.05). Zn induced a reduction of Cd-induced MT-1s expression, in particular of MT-1X (3-fold), and subsequently the final intra-cellular content of Cd. By reducing Cd accumulation in cells, Zn reversed Cd anti-proliferative and anti-inflammatory effects but preserved the low MMP-3/TIMP-1 ratio induced by Cd, which was enhanced by inflammatory conditions.

**Conclusion:**

Zinc counteracts the deleterious effect of Cd by reducing its import and accumulation in the cell, without the reactivation of destructive pathways such as MMPs.

## Introduction

Zinc (Zn) and cadmium (Cd) belong to the group 12 of divalent metals and have similar characteristics, like chemical properties, homeostasis regulation and oxidative stress induction mechanisms [1,2]. Zinc and Cd are mainly transported through the cell using Zrt-Irt-Proteins (ZIPs 1–14) importers, Zn-Transporters (ZnTs 1–10, with ZnT1 as the only membrane exporter) and the metallothioneins (MT-1 and -2), which are cysteine-rich, heavy metal-binding proteins controlling metal homeostasis in cells [3]. Metal regulatory transcription factor (MTF-1) induces MTs transcription, in particular of MT-1s in response to metal exposure, oxidative stress and hypoxia [3–5].

Cadmium has an higher affinity for MT-1s in comparison to Zn and other metals [6]. Cd replaces Zn in Zn-MT bonds without changing their shape and the cellular detoxification action against Cd takes place through MTs. The binding of Cd to MTs is a detoxification mechanism, saturating at high concentrations of Cd. Even if the affinity of Cd for MTs is the strongest, in some models a large supply of Zn has a protective role against the deleterious effects of Cd-exposure [7] through the replacement of Cd by Zn [8]. Zn is necessary for the normal immune response and for cell proliferation [9,10]. In contrast Cd inhibits the activity of anti-oxidative enzymes and the mitochondrial electron transport-chain [11], interferes with metalloproteins [12], inhibits the humoral and cellular immune response at low doses [13,14] and eventually enhances apoptosis or cell proliferation [15,16].

The effects of Zn and Cd were previously studied individually by us and others in the context of rheumatoid arthritis (RA) [17,18]. RA is a chronic inflammatory disease characterized by systemic and local inflammation, where the production of pro-inflammatory cytokines (IL-17, TNF, IL-6) by infiltrated immune cells leads to synovitis and cellular hyperproliferation in the joint. The importance of IL-17 has been demonstrated through the emergence of IL-17-blockade as therapy [19,20] and IL-17 and TNF contribute in synergy to IL-6 and IL-8 production from synoviocytes and other cell types [21,22]. Zn excess was found to contribute to the perpetuation of inflammation by increasing IL-6 secretion from target cells [17]. In contrast, intra-articular administration of Cd at low doses reduced cellular proliferation, inflammation and destruction in an in vivo model of arthritis, by inducing synoviocyte apoptosis [18].

The aim of this work was to evaluate the changes induced by Cd addition on Zn transport and homeostasis, and, as a follow up of our previous work, the regulatory effects of Zn on Cd cytotoxicity by measuring cell viability and inflammation in an in vitro model of RA. Results obtained with RA synoviocytes were compared to those obtained with osteoarthritis (OA) synoviocytes, considered as a less inflammatory synoviocyte phenotype [23,24].

## Materials and methods

### Biopsies, isolation and culture of synoviocytes and co-cultures

Biopsies of synovial tissue samples have been aseptically isolated from RA and OA patients’ joints. The study was approved by the ethics committee of the hospitals of Lyon and patients signed a written informed consent form. The RA patients fulfilled the American College of Rheumatology criteria for RA (18). The synovial tissue was minced into small pieces, which were allowed to adhere to plastic plates. Those samples were maintained in Dulbecco’s Modified Eagle Medium (DMEM, Eurobio, Courtaboeuf, FR) supplemented with 10% FBS (Life Technologies by Thermo Fischer scientific, Grand Island, NY, USA), 2% Penicillin-Streptomycin, 1% L-glutamine and 1% Amphotericin B (all Eurobio) until cells colonized the plastic dishes. At 90% confluence, tissue pieces were removed and cells were trypsinized. Synoviocytes were used between the fourth and ninth passages to ensure the cell specificity. All data are the results of at least three separate experiments (n = 3) using cells taken from three different patients for each type (OA and RA).

### Cytokines, metals and PHA exposure

Synoviocytes and biopsies were pre-exposed overnight to a combination of IL-17A at 50 ng/ml (R&D systems, Minneapolis, MN, USA) and TNF-α at 0.5 ng/ml (R&D systems), prior to metal exposure. The day after Cd alone (0.1 ppm), or Cd and Zn (0.1 ppm 0.9 ppm respectively), or a combination multiple metals were dissolved in nitric acid (0.1 part per million (ppm) of copper (Cu), lead (Pb), indium (In), tin (Sn) and bismuth (Bi), 0.01 ppm of cadmium (Cd) and 0.001 ppm of gold (Au)) were added to the culture medium, in the presence or not of cytokines. To assess the extent of exchange between the cells and the ambient medium, negligible amounts of isotope ^70^Zn were added (0.01ppm).

### Metal fractionation constant between medium and cells, Cd cell content and kinetics by ICP-MS

Synoviocytes (5*10^5^) were cultured with cytokines and metals being optionally added. Supernatants (2ml) and cells were uptake at the end-point (day 14 for metal cocktail and day 5 for Cd only) to analyze metal fractionation constants (K_D_). K_D_ between cells and medium was calculated as K_D_ = (Me/^70^Zn) _cells_/(Me/^70^Zn) _medium_. Values of K_D_ of ~1 indicate an isotopic equilibrium, whereas K_D_>1 indicates that exchange reactions are still ongoing.

To analyze Cd kinetics and cell content, 2ml of supernatant were collected at 6, 12, 24, 48 hours. Cells were than washed with PBS and fresh complete DMEM was substituted for the Cd-enriched medium, to study the possible exit of Cd from cells. Two ml of medium were collected immediately after the wash, at 60, 72, 96 and 120 hours, while cells were collected and counted at the endpoint (120 hours). All the samples were then mineralized with HNO_3_ 0.5N plus H_2_O_2_ (15–20%) at 100°C. Prior the analysis, the mineralized samples were re-dissolved in a 5% HNO_3_ solution in deionized water in an ultrasonic bath. Metal ions in the samples were then measured on a single collector ICP-MS platform ELEMENT 2 (Thermo Finnigan, Ringoes, NJ, USA) which allows metals to be measured at concentrations levels as low as 10^−12^ units, i.e. in the part per trillion (ppt) range.

### Quantification of the gene expression of Zn transporters and metalloprotease-3 by quantitative real-time PCR

OA and RA synoviocytes were plated at a density of 2,5*10^5^ cells/cm^2^ in 12-well plates and were then exposed or not to cytokines overnight followed or not by Cd exposure for 6 hours. After 6 hours of treatment, total RNA was extracted using the RNeasy Mini Kit (Qiagen®, Hilden, GE) and quantified with the Quant-it kit assay (Invitrogen™ by Thermo Fisher Scientific, Grand Island, NY, USA) following manufacturer’s instructions. cDNA was synthesized using the QuantiTect reverse transcription kit (Qiagen®) according to the manufacturer’s instructions. SYBR green-based real time qRT-PCRs were performed on the CFX96 Real-Time PCR Detection System (BioRad, Hercules, CA, USA) using the QuantiFast SYBR green kit and QuantiTect primers (Qiagen®). Cycle threshold values were normalized with respect to the endogenous control gene glyceraldehyde 3-phosphate dehydrogenase (GAPDH). The relative expression of the genes in treated cells versus control cells was determined using the comparative threshold cycle method as described by the manufacturer.

### Cell viability quantification by neutral red assay

The neutral red assay determines the accumulation of the neutral red dye in the lysosomes of viable cells as described by Borenfreund and Puerner (19). Synoviocytes were plated in duplicates at a density of 10^4^ cells/cm^2^ in 96-well plates. After exposure or not to cytokines, metals (Cd alone or Cd plus Zn) and peripheral blood mononuclear cells (PBMCs) (activated or not) for 1, 5 and 8 days, cells were incubated for 150 min with 80 μg/ml of neutral red dye 0.33% (Sigma-Aldrich, St. Louis, MO, USA) at pH 6.5 in serum free DMEM. Cells were then washed with PBS followed by 10 min incubation in 200 μl of elution medium (ethanol/acetone, 50%/1% in deionized water). Absorbance at 540 nm was measured using VICTOR™ X4 plate reader (Perkin Elmer, Waltham, MA, USA) and results were obtained by subtracting the background read at 690 nm and the 540 nm-absorbance in medium without cells.

### Measurement of the production of the pro-inflammatory cytokine IL-6

IL-6 production was quantified in synoviocytes, co-cultures and biopsies supernatants of day 1, 5 and 8 by standard ELISA techniques according to the manufacturer’s instructions (R&D system, San Diego, CA, USA). Absorbance at 450 nm was measured using the VICTOR™ X4 plate reader and results were obtained by subtracting the background read at 540 nm.

### Cell/biopsy imaging

Phase-contrast photographs were taken at day 5 with a Nikon ECLIPSE S100 microscope to observe morphologic changes in cells after exposure to metals.

### Statistical analysis

Data are expressed as the mean ± standard error of the mean (SEM). Statistical significance of changes was determined by GraphPad Prism™ using nonparametric statistical methods. A two-way ANOVA test, followed by multi-parametric analysis, was used in the case of exposure to both metals and cytokines. A Mann-Whitney paired test was used in the case of single exposure, with pairing between same patient’s cells. Differences resulting in p-values inferior or equal to 0.05 were considered statistically significant.

## Results

### Synoviocytes get preferentially Cd-enriched in comparison to Zn and other metals

**Fig 1:**
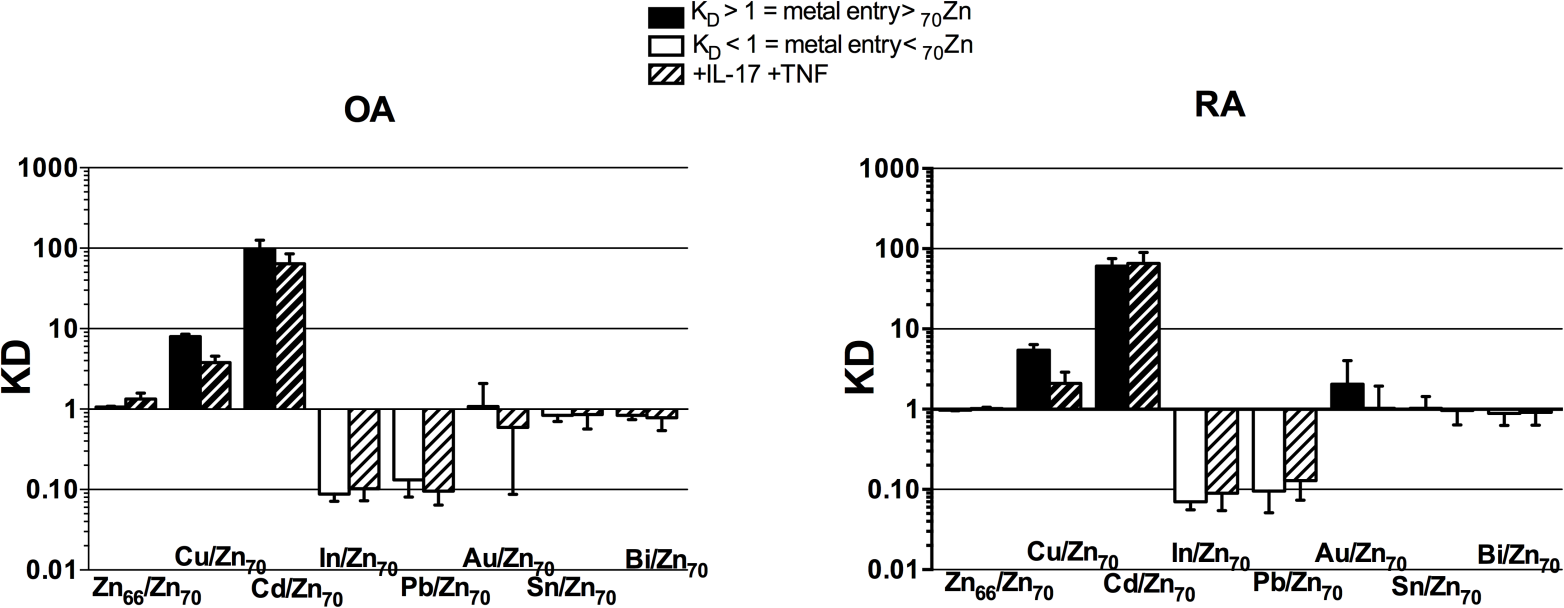
Metal fractionation constant indicates that Cd is preferentially absorbed by synoviocytes. K_D_ = (Me/^70^Zn) _cells_/(Me/^70^Zn) _medium_ (where Me = metal) was calculated at 120h after addition of Cu, Cd, In, Pb, Au, Sn, Bi and ultra-trace amounts of ^70^Zn in cultures of OA or RA synoviocytes, treated (striped bars) or not (normal bars) with IL-17 and TNF-α. At equilibrium, K_D_ = 1. Black lines represent the high affinity group, while white bars the low affinity group. Data are the mean of at least three independent experiments.

Synoviocytes were exposed to a cocktail of indium (In), tin (Sn), lead (Pb), bismuth (Bi), copper (Cu), cadmium (Cd) and gold (Au), to measure the relative affinity of these metals for the intracellular medium. Zinc was added to cultures in a ‘tracerless’ form using minute quantities of a stable ^70^Zn spike, a very minor natural Zn isotope (0.6% of the total of the other isotopes), which admits a correct follow-up of metals through the cells without noticeably modify the amount of Zn present in the culture. The affinity ratios (*K*_D_) of metals between cells and medium were referred to ^70^Zn isotope as tracer (see materials and methods). A Zn *K*_D_ value near 1 unity (isotopic equilibrium) indicates that the proportion of all isotopes is the same in the cell and the medium, and therefore that cells efficiently exchanged Zn with the medium (Fig 1A). The metals could be divided into three groups according to their relative affinity with respect to Zn. The high-affinity group was made of Cu and Cd. The *K*_D_ for Cu was 6–8 in the presence or absence of cytokines in both OA and RA synoviocytes. *K*_D_ for Cd was 10 fold stronger, reaching 70–100, which was at the low end of literature values [25,26]. The low-affinity group, made of In and Pb, had a *K*_D_ <1, which indicated that the cells efficiently discriminated against these metals. The indifferent group (Au, Sn, Bi), with a *K*_D_~1, suggested that metal importers did not recognize and treat them as being different from Zn (Fig 1A). This larger affinity of Cd for cellular import was in line with previous data describing rapid Cd enrichment in inflammatory synoviocytes [18]. The following part of the work will focus on the effects of Zn on Cd modified homeostasis in the inflammatory context of rheumatoid arthritis, with interest given to Cd/Zn shared transport.

### Zn excess modulates Cd kinetics and reduces final Cd intra-cellular content

**Fig 2:**
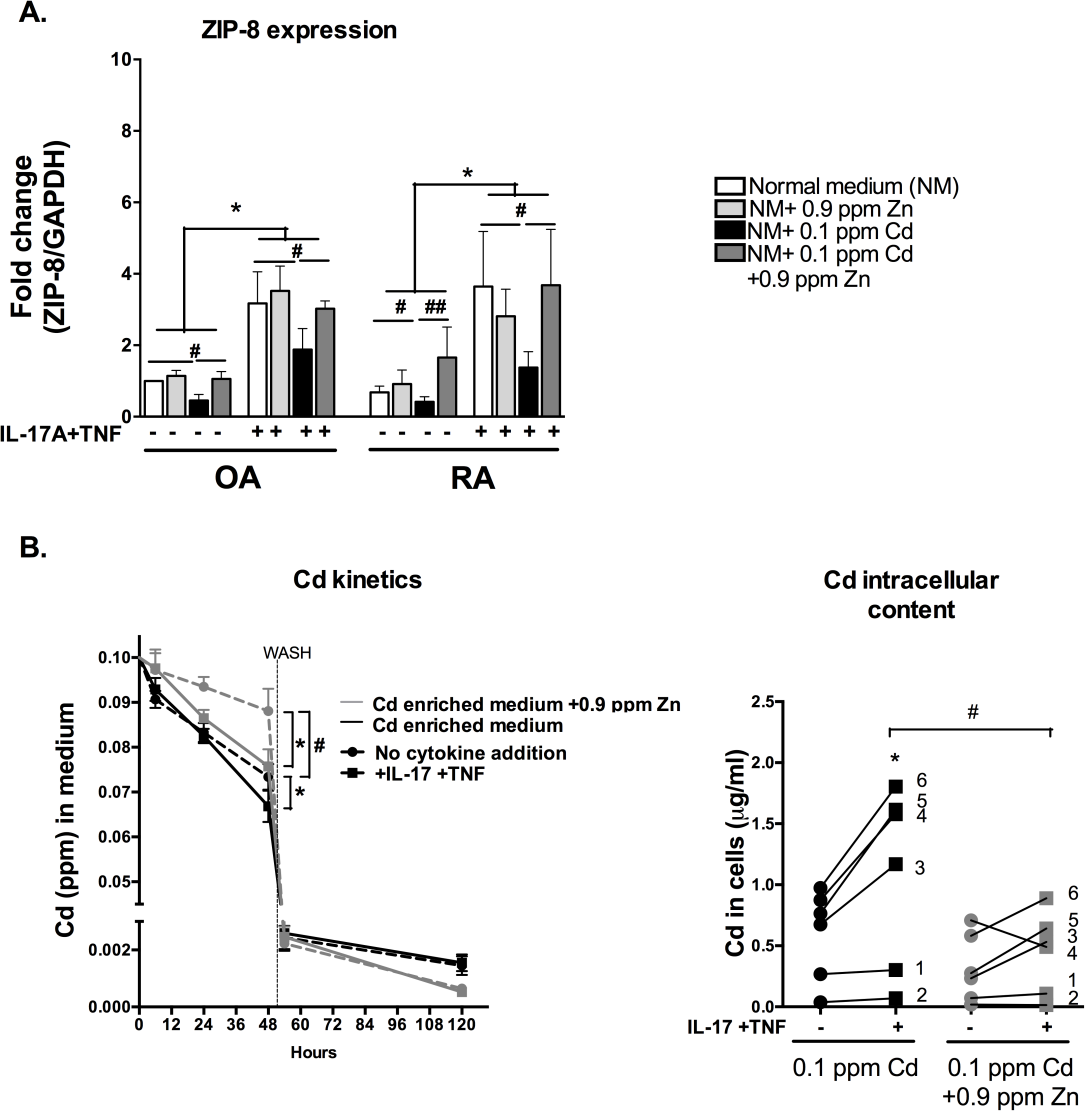
Zn excess modulates Cd kinetics and reduces final Cd intra-cellular content, despite ZIP-8 overexpression. (A) The gene expression of the importer ZIP-8 importer was quantified after overnight exposure to IL-17A and TNF-α alone or in combination. Results are normalized with GAPDH expression and are presented as fold changes compared to control. (B) In the left panel Cd concentration in the RA synoviocyte culture medium as a function of time (kinetics). Dashed line: control experiment with no cytokine addition. Solid line: cytokines added to the culture medium. On the right panel Cd uptake calculated from Cd concentration measured in the cells expressed in ng per 10^6^ cells at time t = 120 hours. Data are the mean of at least three independent experiments. Data are presented as mean ± SEM; */# p<0.05, **/## p<0.01, * refers to the analysis of cytokine exposure and # refers to the analysis of metal exposure.

The expression of the constitutive membrane Zn/Cd importer ZIP-8 is sensitive to inflammation as already described in synoviocytes [17] or in other cells (such as monocytes, macrophages or epithelial cells) [27]. Herein, the addition of IL-17 and TNF combined enhanced the expression of ZIP-8 in both OA or RA cells and metal conditions (Zn addition, Cd addition, or both). The addition of Zn alone (0.9 ppm) or Cd plus Zn (0.1 ppm and 0.9 ppm respectively) combination did not change the expression of ZIP-8. Conversely the addition of Cd alone for 6 hours reduced the expression of ZIP-8, in comparison to the control condition (Fig 2A), with or without cytokine stimulation, with a possible impact of Cd toxicity.

Cd kinetics from the extra-cellular to the intra-cellular medium was increased by IL-17/TNF treatment, leading to an increased Cd content in cells after 120 hours (p<0.05),as previously described [18]. Cytokine treatment enhanced Cd absorption even in the presence of an excess of Zn (0.9ppm) (p<0.05). In the absence of cytokines, the absorption rate from the medium was lower when Zn excess was added to the culture medium in comparison to the Cd alone condition (~20% greater Cd cellular absorption from the medium in the presence of Cd alone, p<0.05). In conclusion, Zn excess modified the kinetics of Cd in synoviocytes resulting in a reduced intracellular accumulation of Cd in comparison to Cd-alone condition (Fig 2B). Thus, increasing Zn by a factor 3 relative to the standard culture medium concentration reduced the entry of Cd into the cells via ZIP-8.

### Zn addition to the Cd-enriched medium reduces MT-1s expression

MT-1s have a major role in cell detoxification from heavy metals like Cd, while MT-2s mainly participate to the regulation of Zn homeostasis [28]. Moreover, MT-2s are overexpressed in the injured cartilage in OA, inducing chondrocyte apoptosis and matrix degradation through the metalloproteases (MMPs) MMP-3 and 13 [29]. MMP-3 has an important role in the invasive phenotype of RA synoviocytes [30–32].

**Fig 3:**
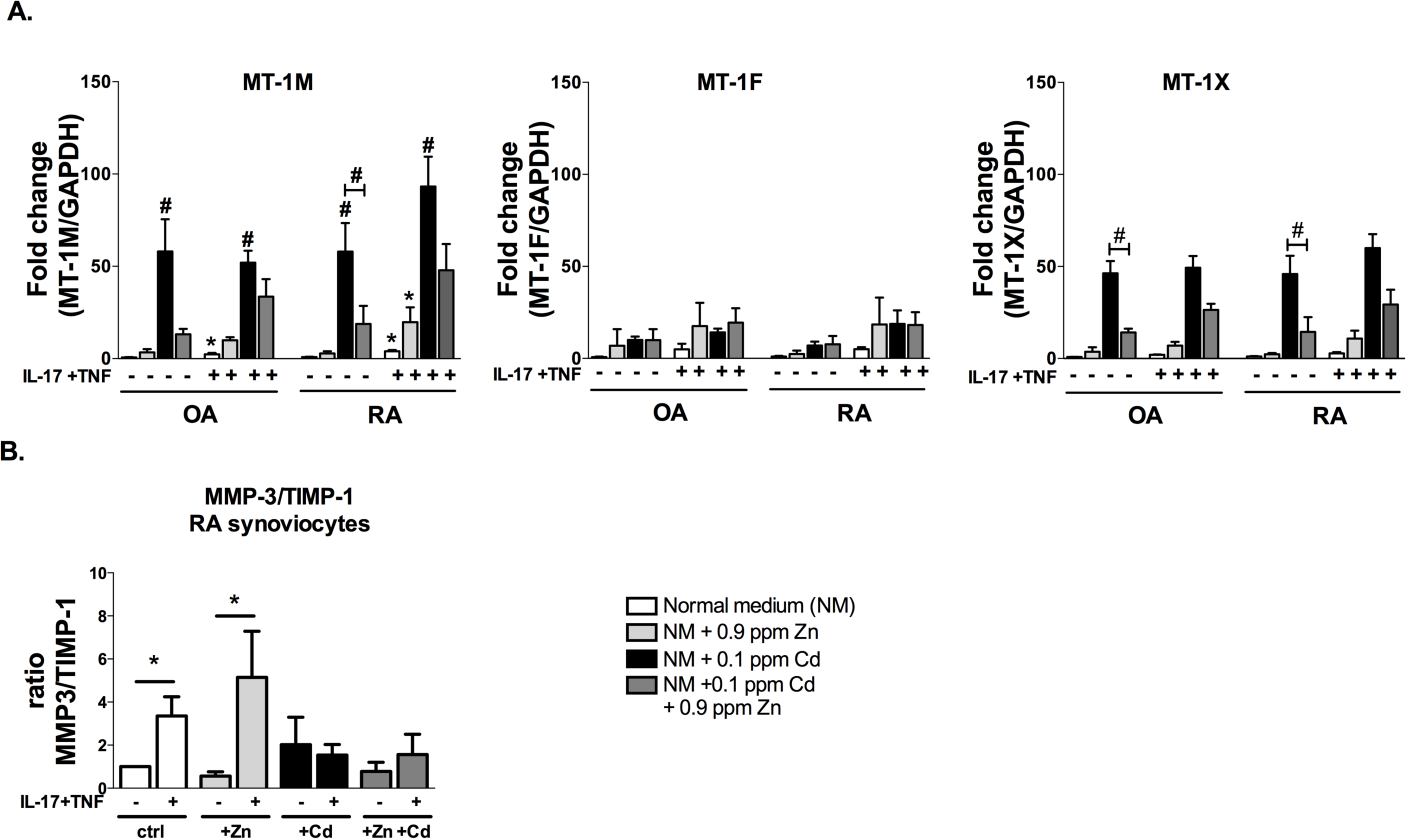
Zn addition in the Cd-enriched medium reduced MT-1s expression without effect on Cd-reduced MMP-3/TIMP-1 ratio. (A) The gene expression MT-1s and (B) the ratio MMP-3/TIMP-1 were quantified by q-RT-PCR in synoviocytes under control conditions and in synoviocytes exposed overnight to a combination of recombinant IL-17A (50ng/mL) and TNF-α (0.5ng/mL) in the presence of Zn, or Cd, or the combination of both. Results are normalized with GAPDH expression and are presented as fold changes compared to control. Data are the mean of at least three independent experiments. For data in which the effects of both cytokines and Zn are assessed, a two-way Anova test was used (A). For data for which only the effect of cytokines is assessed, a Mann-Whitney test was used (B). Data are presented as mean ± SEM; */# p<0.05, **/## p<0.01, * refers to the analysis of cytokine exposure and # refers to the analysis of metal exposure.

Regarding the regulation of MT-1 expression, Zn alone did not induce significant changes in MT-1s expression while Cd addition induced a 50-fold increase of the expression of MT-1Mand MT-1X, and to a lesser extent of MT-1F, in both synoviocyte types. The simultaneous exposure of synoviocytes to Zn and Cd resulted in a reduced expression of MT-1s relative to cells exposed to Cd-only (Fig 3A). This reduction was stronger for MT-1X expression, where the control-relative Cd-induced MT-1X overexpression was reduced after Zn addition, in both RA (fold change mean ± SD 45.95 ± 9.90 vs.14.46 ± 8.00; p<0.05) and OA (fold change mean ± SD 49.29 ± 6.41 vs. 26.42 ± 3.29; p<0.05) cells. MT-1M expression was significantly reduced by the simultaneous exposure to Cd and Zn in RA cells compared to the Cd-alone condition (reduced fold change from mean ± SD 57.95 ± 15.53 to mean ± SD 18.75 ± 9.77, p<0.05), only in cytokine unstimulated RA cells. The expression of MT-2 was measured after exposure to metals and cytokines, showing no significant changes between conditions, but only a tendency after cytokine addition (S1 Fig).

In normal tissue (i.e. cartilage, synovial tissue) a well-established balance exists between active MMP levels and levels of their inhibitors TIMP. An imbalance between MMP-3 and TIMP-1 has been observed in inflammatory conditions such as RA, with IL-17/TNF contributing in synergy to the increased MMP/TIMP ratio [30–32].The possible increase of MMPs/TIMP-1 ratio in association with the increased level of MT1s after exposure to cytokines and metal was measured. As expected, IL-17 and TNF synergistically enhanced the MMP-3/TIMP-1 expression ratio. The addition of Zn did not modify the effect of cytokines, while the addition of Cd reversed the IL-17/TNF induced increase of the MMP-3/TIMP-1 ratio. The addition of an excess of Zn (0.9 ppm) to Cd-enriched medium was not sufficient to change the effect of Cd alone on MMP-3/TIMP-1 ratio (Fig 3B).

The Zn excess reduced Cd-uptake from synoviocytes and down-regulated the expression of metal homeostasis regulators MT-1s, with particular impact on MT-1X and MT-1M. Thus, Cd, but not Zn, was the limiting factor for MT-1 expression, in line with the role of MT in cell detoxification. Moreover, Cd addition correlated with a reduced MMP-3/TIMP ratio, previously associated with low destruction and a reduced inflammation, in line with the observations made in the in vivo model of arthritis after intra-articular Cd injections [33]. No changes were observed on the MMP-3/TIMP ratio.

### Zinc reverses the anti-proliferative and anti-inflammatory effects of Cd on synoviocytes

**Fig 4:**
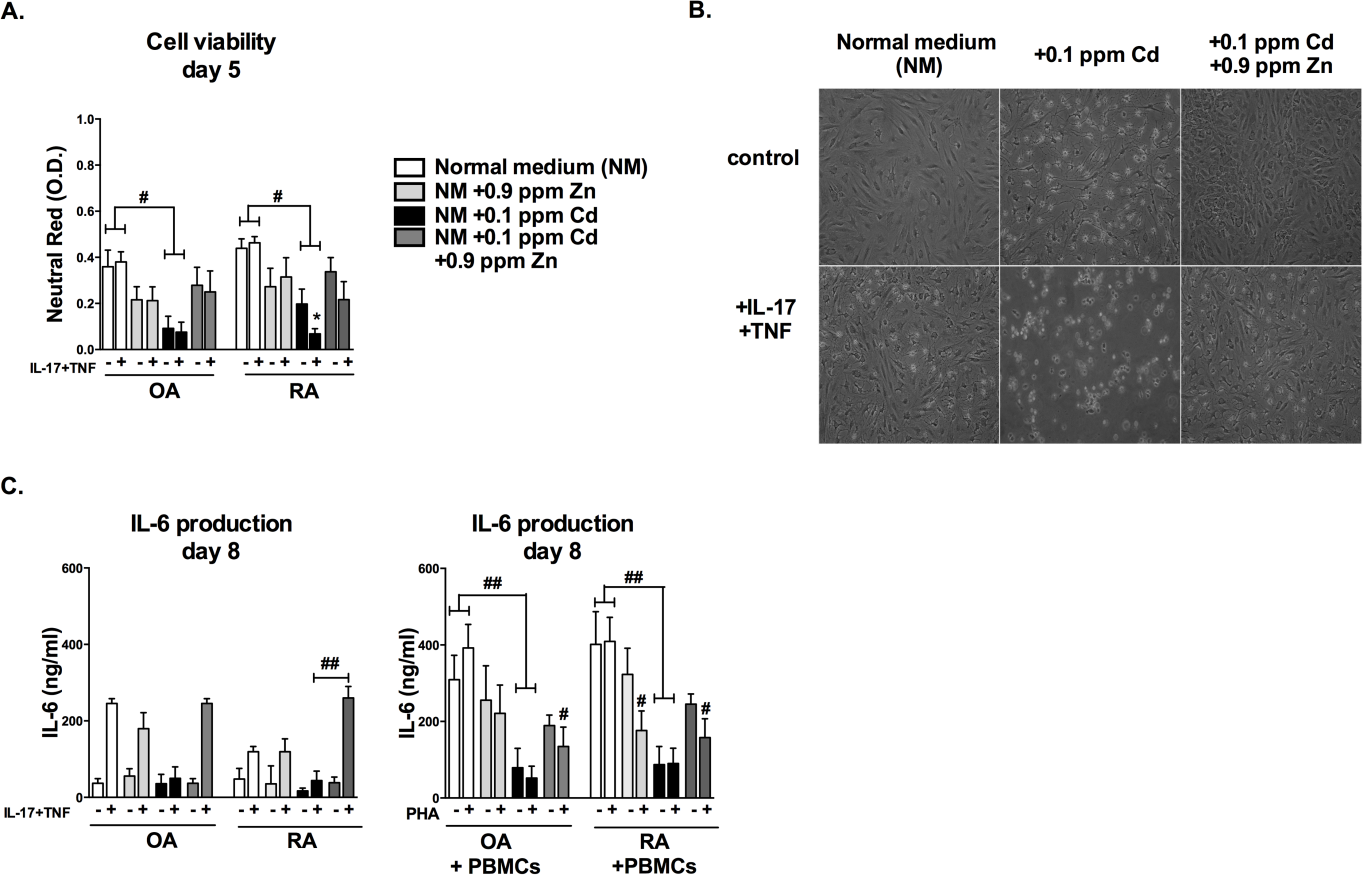
Zinc reverses the anti-proliferative and anti-inflammatory effects of Cd on synoviocytes. (A,B) OA and RA synoviocytes were treated overnight with IL-17A (50 ng/mL) and TNF-α (0.5 ng/mL) followed by the addition of Zn, Cd or the combination of both. Cell viability and phase contrast microscopy photographs were assessed at day 5. (C) OA and RA synoviocytes were treated in normal cultures as in A or in co-cultures with inactivated or PHA-activated PBMCs. Supernatants were collected at day 8 and IL-6 production was quantified by ELISA. Statistical significance was assessed by a two-way Anova. Data are presented as mean ± SEM; * refers to the analysis of cytokine exposure, # to the analysis of metal exposure */# p<0.05, **/## p<0.01.

Competition between Zn and Cd accounts for the protective role of Zn against Cd toxicity [7,34,35]. Zn addition in the medium did not modify synoviocyte viability in comparison to the control situation. Conversely, Cd strongly reduced cell viability and this effect was enhanced in the presence of IL-17 and TNF combination [18]. Zn was therefore added to Cd-treated cultures to clarify its possible enhancing or competitive effect on cell viability and inflammation. Zn addition partially reversed the effect of Cd on synoviocyte viability, reducing Cd-induced synoviocyte detachment from the plate [36]. At day 5, the viability score of Cd-Zn treated cells was twice that of Cd-only treated cells, whether cytokines were present or not (Fig 4A, p<0.05). The combined addition of IL-17/TNF-α plus Cd increased this effect (Fig 4B). Reduction of Cd-induced synoviocyte death upon Zn addition (0.9 ppm, representing 3-fold the classic DMEM medium amounts) was clearly visible in phase contrast microscopy at day 10 (pictures Fig 4B). Moreover, exposure to Zn reduced the effects of Cd on IL-6 production in both cultures of synoviocytes and co-cultures of synoviocytes with PBMCs (Fig 4C). In the presence of cytokines, RA synoviocytes production of IL-6 was reduced from (mean ± SD) 120 ± 14 ng/ml without Cd to (mean ± SD) 44 ± 25 ng/ml with Cd (p<0.01). In contrast, RA synoviocytes exposed to Cd and Zn combined in the presence of cytokines produced a far more abundant quantity of IL-6 (mean ± SD) 260 ± 30 ng/ml in Cd Zn exposed synoviocytes vs. 44 ± 25 ng/ml in Cd only exposed synoviocytes, p<0.01). Moreover, the addition of Zn to RA synoviocyte cultures treated with Cd not only restored the production of IL-6 in comparison to Cd-only treated cells, but increased IL-6 production over the control situation (46% increase over the control production). The IL-6 production by OA synoviocytes could also be restored to the control situation by Zn addition (Fig 4C, left panel). Likewise, the production of IL-6 was measured in co-cultures of synoviocytes and PBMCs to reproduce the cell interactions at the site of inflammation. Zinc alone slightly reduced IL-6 production in comparison to control in RA synoviocytes cultured with PHA-activated PBMCs (mean ± SD 409 ± 114 ng/ml in the RA control versus mean ± SD 152 ± 99 ng/ml in Zn treated PHA activated RA co-cultures, p<0.05). In this co-culture model, Cd reduced the production of IL-6 of 75% to the lesser extent in both OA and RA co-cultures, independently of PHA-activation. The addition of Zn and Cd combined restored only partially IL-6 production in the non-activated co-culture, resulting in a final 40% inhibition of IL-6 production in both OA and RA co-cultures. In activated co-cultures, the co-addition of Zn to Cd co-cultures was not sufficient to completely restore the IL-6 production, still resulting in a significant reduction (65% inhibition) of IL-6 production in comparison to the activated control co-cultures (Fig 4C, right panel).

## Discussion

Zinc and Cd use the same transport mechanisms for the entry and the homeostasis regulation in cells. In the inflammatory context of arthritis, Zn supplementation induces a feedback loop on IL-17/TNF induced inflammation and results in an overexpression of the importer ZIP-8 and MT-1s [17]. Conversely Cd, which is imported through the same mechanisms, induces synoviocyte apoptosis, strongly reducing the release of IL-6 [18]. Zinc metabolism impacts Cd-induced toxicity in synoviocytes as Cd uses Zn transporters to enter the cells and replaces Zn in MTs. This process is described as the detoxifying role of MTs against Cd toxicity [37] The effect of Cd addition on physiological Zn transport and homeostasis, as well as the regulatory effect of Zn on Cd-induced cytotoxicity was evaluated in this study, as a follow-up to our previous study using RA synoviocytes pre-exposed or not to IL-17/TNF induced-inflammation. Here a far more abundant supply of Zn interfered with Cd import and could counteract to its deleterious effects.

First, the ability of a wide range of metals to enter synoviocytes was compared to that of ^70^Zn, resulting in a selective and higher entry of Cd in synoviocytes with respect to Zn and other metals. The K_D_ of Cd between cells and culture medium was found to be further enhanced by inflammation in RA synoviocytes only. This result implies a different response to inflammation of OA and RA synoviocytes, with possibly a different modulation of metal transporters.

ZIP-8 expression is mainly up-regulated by inflammation in both OA and RA synoviocytes, in particular with a synergistic effect of IL-17 and TNF on RA synoviocytes [17]. The addition of Cd alone in synoviocyte culture medium negatively modulated ZIP-8 expression as previously described in other cell types [38], while Zn addition to Cd restored it to the control situation. Addition of Cd reduced inflammation-dependent up-regulation of ZIP-8 expression, but not the final cellular Cd content, which was even higher in the presence of Cd alone than Cd plus Zn. Thus, Cd accumulation could be driven by transporters other than ZIP-8, as described in the literature [39]. ZIP-8 is a high-affinity Cd transporter expressed in synoviocytes together with NRAMP-2, also known as DMT1 [40]. The mRNA expression of DMT-1 in RA synoviocytes, herein was not modulated by inflammation or by Cd-exposure (S1 Fig). Other channels mediate Cd uptake in synoviocytes with a lower affinity, as it is the case for TRP calcium channels and in particular TRPV1 and TRPV4 [41]. These channels respond to a TNF stimulus [42] and the effect of the synergy of IL-17 and TNF remains to be studied. Moreover the study of the effects of the anti-inflammatory cytokines (IL-10, TGFβ) could provide more specific information on the type of microenvironment needed for enhancing Zn transporter expression.

The reduced Cd transport in the presence of Zn is in line with their competitive action for ZIP-8. ZIP-8 is not selective between divalent metals, except for Cu which is not transported, but its expression is only dependent on inflammation and iron availability by post-transcriptional mechanisms [43].

Addition of Zn to the Cd-treated cultures could partially reverse the effects of Cd on synoviocytes as cell viability was increased and the production of IL-6 restored. The results of the present study are in agreement with published reports, demonstrating the protective effect of Zn in preventing Cd-induced local or systemic alterations [44,45]. Moreover, MT-1 levels were reduced in the presence of 0.9 ppm Zn and 0.1 ppm Cd. This result indicated that MT-1 expression was reduced because of its Cd specificity, indicating a higher specificity of MTs for Cd in comparison to other transporters, i.e. ZIP8. The increased ratio MMP-3/TIMP-1 ratio indicated an enhanced activity of metalloproteases in the presence of inflammation which could be reduced by Cd and was not modified by addition of Zn. The data obtained on the MMP/TIMP pathway could be verified by intra-articular injection of Zn in the rat model following Cd therapeutic treatment.

In conclusion, there is a continuous flux of Zn through the cell and inflammation increases Zn cellular import and storage, as previously anticipated by our mathematical model [17]. When Cd is added to an inflammatory experimental condition, it is also imported. The presence of an excess of Zn can reduce the import of Cd and can protect the cell from the deleterious effect of Cd, as the two metals compete for the same import pathway. The cell probably uses Zn and Zn-trafficking molecules as a detoxifying tool against Cd, as the addition of Zn reduces the apoptotic effect of Cd resulting in a higher production of IL-6. The use of Cd as a potent inducer of synoviocyte apoptosis by intra-articular injection [18], could be modified by the circulating and intra-articular concentration of Zn in patients. Zn excess or deficiency could change the Cd concentration outside of the therapeutic range.

## Supporting information

S1 Fig Inflammation and metal addition did not significantly modify DMT-1 and MT-2 expressions. The gene expression of DMT-1 and (B) of MT-2 were quantified by q-RT-PCR in RA synoviocytes under control conditions and in synoviocytes exposed overnight to a combination of recombinant IL-17A (50ng/mL) and TNF-α (0.5ng/mL) in the presence of Zn, or Cd, or the combination of both. Results are normalized with GAPDH expression and are presented as fold changes compared to control. Data are the mean of at least three independent experiments. For data in which the effects of both cytokines and Zn are assessed, a two-way Anova test was used (A). For data for which only the effect of cytokines is assessed, a Mann-Whitney test was used (B).
(TIFF)
