# Supplementary figures and images for: Regulatory effects of zinc on cadmium-induced cytotoxicity in chronic inflammation
Source: PLoS One. 2017 Jul 25;12(7):e0180879. doi: 10.1371/journal.pone.0180879 (PMC5526586; doi:10.1371/journal.pone.0180879)

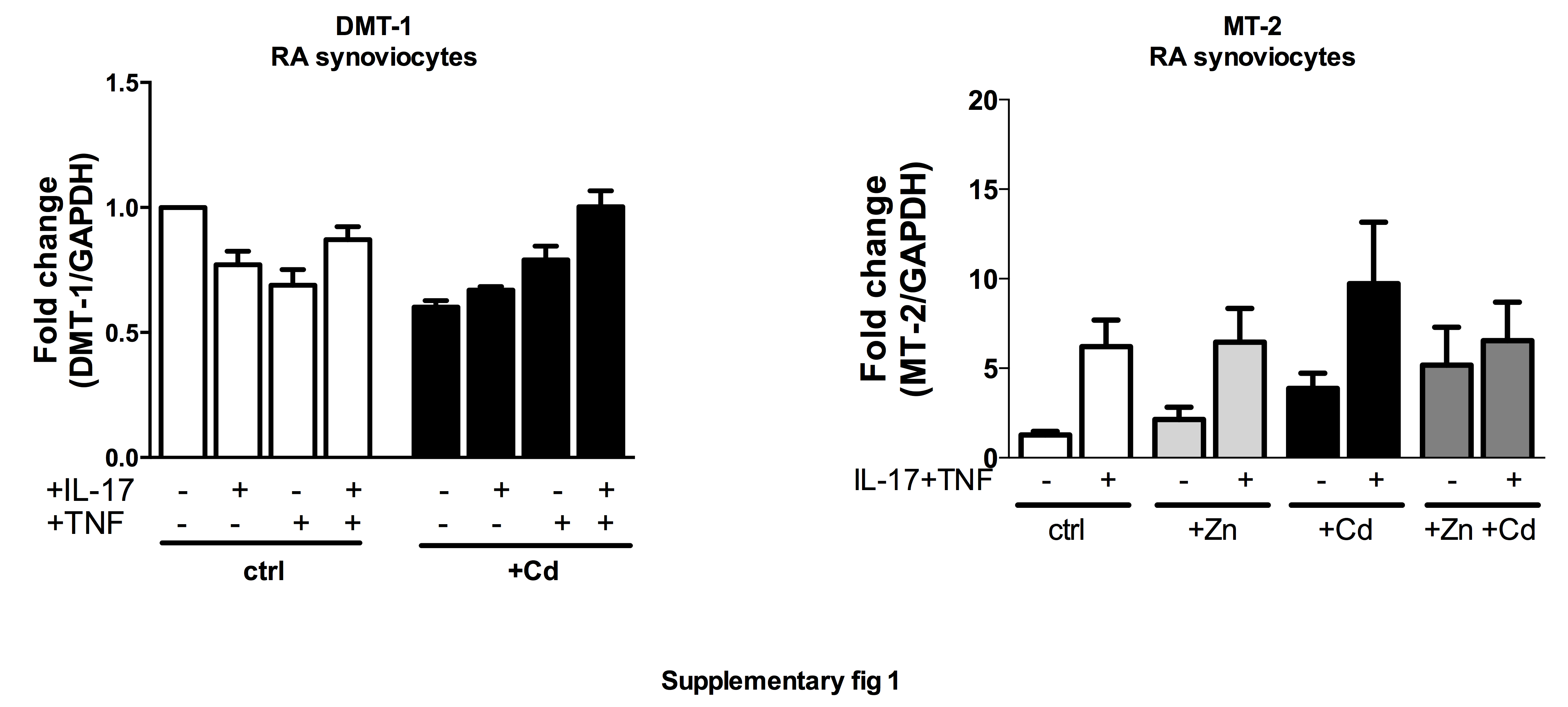

Supplement: S1 Fig — The gene expression of DMT-1 and (B) of MT-2 were quantified by q-RT-PCR in RA synoviocytes under control conditions and in synoviocytes exposed overnight to a combination of recombinant IL-17A (50ng/mL) and TNF-α (0.5ng/mL) in the presence of Zn, or Cd, or the combination of both. Results are normalized with GAPDH expression and are presented as fold changes compared to control. Data are the mean of at least three independent experiments. For data in which the effects of both cytokines and Zn are assessed, a two-way Anova test was used (A). For data for which only the effect of cytokines is assessed, a Mann-Whitney test was used (B). (TIFF) [file pone.0180879.s001.tiff]
